# Barriers and facilitators to the implementation of antenatal syphilis screening and treatment for the prevention of congenital syphilis in the Democratic Republic of Congo and Zambia: results of qualitative formative research

**DOI:** 10.1186/s12913-017-2494-7

**Published:** 2017-08-14

**Authors:** Dalau Nkamba, Musaku Mwenechanya, Arlette Mavila Kilonga, Maria Luisa Cafferata, Amanda Mabel Berrueta, Agustina Mazzoni, Fernando Althabe, Ezequiel Garcia-Elorrio, Antoniette K. Tshefu, Elwyn Chomba, Pierre M. Buekens, Maria Belizan

**Affiliations:** 1 Ecole de Santé Publique de Kinshasa, Kinshasa, Democratic Republic of Congo; 2 University Teaching Hospital of Lusaka, Lusaka, Zambia; 3 Institute for Clinical Effectiveness and Health Policy (IECS), Buenos Aires, Argentina; 4 Kinshasa School of Public Health, University of Kinshasa, Kinshasa, Democratic Republic of Congo; 5 University Teaching Hospital, Lusaka, Zambia; 6 School of Public Health and Tropical Medicine, Tulane University, New Orleans, USA

**Keywords:** Congenital syphilis, Syphilis screening, Formative research, Qualitative research

## Abstract

**Background:**

The impact of untreated syphilis during pregnancy on neonatal health remains a major public health threat worldwide. Given the high prevalence of syphilis during pregnancy in Zambia and Democratic Republic of Congo (DRC), the Preventive Congenital Syphilis Trial (PCS Trial), a cluster randomized trial, was proposed to increase same-day screening and treatment of syphilis during antenatal care visits. To design an accepted and feasible intervention, we conducted a qualitative  formative research. Our objective was to identify context-specific  barriers and facilitators to the implementation of antenatal screening and treatment during pregnancy.

**Methods:**

Qualitative research included in-depth semi-structured interviews with clinic administrators, group interviews with health care providers, and focus groups with pregnant women in primary care clinics (PCCs) in Kinshasa (DRC) and Lusaka (Zambia).

**Results:**

A total of 112 individuals participated in the interviews and focus groups. Barriers for the implementation of syphilis testing and treatment were identified at the a) system level: fragmentation of the health system, existence of ANC guidelines in conflict with proposed intervention, poor accessibility of clinics (geographical and functional), staff and product shortages at the PCCs; b) healthcare providers’ level: lack of knowledge and training about evolving best practices, reservations regarding same-day screening and treatment; c) Pregnant women level: late enrollment in ANC, lack of knowledge about consequences and treatment of syphilis, and stigma. Based on these results, we developed recommendations for the design of the PCS Trial intervention.

**Conclusion:**

This research allowed us to identify barriers and facilitators to improve the feasibility and acceptability of a behavioral intervention. Formative research is a critical step in designing appropriate and effective interventions by closing the “know-do gap”.

**Electronic supplementary material:**

The online version of this article (doi:10.1186/s12913-017-2494-7) contains supplementary material, which is available to authorized users.

## Background

Despite several advances in treatment and management, syphilis remains a major public health problem worldwide. Nearly 1.5 million pregnant women are infected with syphilis each year [1], and in Africa it was estimated that the prevalence of syphilis among antenatal care (ANC) attendees was 2.2% in 2013 [2]. In the Democratic Republic of the Congo (DRC), the prevalence of syphilis among ANC attendees was 3.3% [3] whereas it was 4% in Zambia [4]. It is estimated that half of all pregnant women with untreated syphilis will experience adverse birth outcomes such as miscarriage, stillbirth, preterm delivery, low birth weight, neonatal death, and neonatal infection [1, 5, 6].

In order to prevent these outcomes, the World Health Organization (WHO) recommends universal syphilis screening, and treatment of pregnant women testing positive, as routine practice during ANC [7, 8]. Administering a single dose of long-acting penicillin prevents congenital syphilis. Either one (primary or secondary disease) or three (latent disease) penicillin doses are effective to treat maternal syphilis [9].

However, several of key barriers to implementation of universal prevention of congenital syphilis screening have been identified. These barriers range from patient-level factors (e.g., late entry into ANC, costs borne by patients, and the need to return at a later time for results) to facility-level constraints (e.g., health worker absence or insufficient training; provider fears around side effects of benzathine penicillin treatment despite their rarity; and use of a diagnostic procedure that requires infrastructure that facilities may not have consistent access to such as laboratory capacity, cold storage, and electricity) and system-level issues (e.g., low prioritization of syphilis screening and treatment by health policy makers) [10–12].

In accordance with theories of implementation [13, 14], the intervention proposed by the Preventing Congenital Syphilis Trial (PCS Trial) (see Additional file 1: Appendix S1) was developed specifically to target barriers to implementing best practices identified in the literature, including those mentioned above [15]. As such, the intervention consists of the use of point-of-care rapid syphilis diagnostic tests (RDTs) in pregnant women and same-day treatment of those who screen positive with one dose of benzathine penicillin 2.4 MU, plus a behavioral intervention based on theories of health behavior change [16–18]. Specifically, the behavioral intervention consists of (1) identification and training of opinion leaders among ANC providers and (2) packaging the RDTs and pre-measured dosages of benzathine penicillin, erythromicine, anaphylaxis treatment together into ready-to-use syphilis diagnosis and treatment kits. The opinion leaders will be tasked with disseminating, implementing, and maintaining best practices among their peers using reminders, routine monitoring and feedback (Additional file 1: Appendix S1).

In addition, to ensure that the trial strategy would be effective, culturally appropriate, and acceptable to women and ANC providers, we conducted qualitative formative research to assess women’s and providers’ perspectives. The formative research aimed to identify factors that may hinder (barriers) or enable (facilitators) the syphilis screening at first ANC visit and to provide treatment to positive women at the same visit in order to identify additional, context-specific factors that may influence the implementation of PCS Trial intervention. Based on those findings we analyzed the feasibility and acceptability of the proposed intervention. In this paper, we present the results of the qualitative research and recommendations to tailor the intervention.

## Methods

### Study design and setting

The study used inductive inquiry consistent with the grounded theory approach [19] and was conducted in accordance with qualitative research guidelines [20, 21]. It included in-depth semi-structured interviews with clinic administrators, group interviews with health providers (groups were natural groups) [22], and focus groups with pregnant women. It was performed from July to September 2015 during the preparatory phase of the PCS Trial.

Key informants were recruited in 11 of the 26 primary care clinics (PCCs) participating in the PCS trial. Clinics were purposively selected to cover all types of PCCs that will be included in the PCS Trial. In the DRC, we included 8 clinics (2 public, 2 private not-for-profit, and 4 for-profit private) located in the eastern part of Kinshasa, where seven of wich had already implemented the Prevention of Mother to Child Transmission of HIV (PMTCT) program. In Zambia, where all the clinics participating in the PCS Trial are public and have implemented the PMTCT program, we included three clinics.

### Participants and sampling

Interview and focus group participants were purposively-selected to cover three target groups: clinic administrators, health care providers, and pregnant women.

#### Clinic administrators and health care providers

In Kinshasa, we conducted five in-depth semi-structured interviews with individuals responsible for overseeing the clinics, henceforth referred as “clinic administrators”, and eight group interviews with health care providers (henforce “providers”; 4–5 participants per group). In Lusaka, we conducted three group interviews with health care providers (4–5 participants per group). Clinic administrators were not included in Lusaka. All providers present at the moment of the clinic visit were invited and all agreed to participate. The providers ranged from physicians, nurses, midwives, lab technicians responsible for testing pregnant women, counselors (trained in Psychosocial and HIV counseling, and responsible for patient counseling on HIV and other Sexually Transmitted Infections, including Syphilis), and nutritionists (See Table 1).

**Table 1:** Characteristics of the participants, by country

	Zambia	DRC	Total
Characteristics of study participants	• N of PCCs = 3	• N of PCCs = 8	• N of PCCs = 11
	• N of authorities = 0	• N of authorities = 5	• N of authorities = 5
	• N of Providers = 13	• N of Providers = 37	• N of Providers = 50
	• N of Pregnant women = 13	• N of Pregnant women = 44	• N of Pregnant women = 57
PCCs Authorities (Individual Interviews)	0	5	5
Female	0	2	2
Male	0	3	3
Health Providers	13	37	50
Female	8 (62%)	14 (38%)	22(44%)
Male	5 (38%)	23 (62%)	28(56%)
Age Median	40 (32–45)	38 (22–65)	
Speciallity			
Midwife and nurse	4 (31%)	29 (78%)	33(66%)
Physician	1 (8%)	0	1(2%)
Lab Technician	3 (23%)	8 (22%)	11(22%)
Counselors	4 (31%)	0	4(8%)
Nutritionist	1 (8%)	0	1(2%)
Pregnant women (Group Interviews)	13	44	57
Age Median	24 (20–26)	22 (17–41)	
Parity:			
Primiparous	4 (31%)	27 (61%)	31(54%)
Multiparous	9 (69%)	17 (39%)	26(46%)
Education			
Elementary school incomplete	1 (8%)	1 (2%)	2(4%)
Elementary school completed	1 (8%)	3 (7%)	4(7%)
High school incomplete	6 (46%)	23 (52%)	29(51%)
High school completed	1 (8%)	16 (36%)	17(30%)
University	4 (31%)	1 (2%)	5(9%)

Questionnaires for semi-structured interviews and group interviews were developed based on the study objectives and included the following themes: (1) routine process during first ANC visit, (2) availability of resources for syphilis screening and treatment, (3) implementation of policies regarding ANC and prevention of congenital syphilis, (4) providers’ knowledge and skills to test and treat, (5) providers’ attitudes towards syphilis test and treatment, and (6) acceptability of the PCS Trial intervention. (See Additional file 2: Appendix S2 and Additional file 3: Appendix S3).

#### Pregnant women

In Kinshasa, we conducted eight focus groups with pregnant women (4–8 participants) in eight clinics. In Lusaka, we conducted three focus groups with pregnant women (4–5 participants) in three clinics (See Table 1). Focus group were conducted with women attending their first ANC visit. Health facility staff assisted in identifying potential study participants. Snacks and beverages were offered to participants.

The focus group questionnaire was developed based on the study objectives, and included the following themes: (1) acceptability of syphilis screening and treatment at the first ANC visit, (2) knowledge about syphilis and prevention of mother-to-child transmission, and (3) current level of participation in the decision making for clinical procedures during pregnancy including syphilis testing and treatment. (See Additional file 4: Appendix S4).

### Procedure

Researchers trained in qualitative methods were appointed in Kinshasa by the DRC-based research team to conduct the data collection. In Lusaka, data collection was managed by researchers from the trial coordination institution (Institute for Clinical Effectiveness and Health Policy - IECS) and a trained local researcher. The protocol, data collection guidelines, and data analysis strategy were agreed upon by all researchers involved in data collection and analysis.

Interviews and focus groups lasted between 40 and 60 min. In Kinshasa, interviews with professionals were conducted in French and focus groups with women attending ANC were conducted in Lingala. In Lusaka, two interviews with health providers were conducted in English by a researcher from IECS, and the rest (both health providers and pregnant women) were conducted in a local language (either Nyanja or Bemba) by local researchers.

### Data collection

All interviews and focus groups were audiotaped and subsequently transcribed in preparation for analysis. In the DRC, the transcripts were analyzed in French and Lingala, while in Zambia, transcripts were translated into English for the analysis.

### Data analysis

The written transcripts were entered into ATLAS.ti version 7 (2013) qualitative data management software (Scientific Software Development, Berlin, Germany) and were coded according to a codebook based on themes included in the questionnaires and supplemented by a grounded theory-based approach to capture emergent themes [19].

Thematic analysis was done for each target group. Then matrices were developed to facilitate comparisons across the transcript materials and to retain the context of the data (i.e. sites, clinic, and type of informant). Finally, data was abstracted and interpreted. As part of this analysis, direct quotations representative of the participants’ opinions were selected and are included in this manuscript to illustrate the findings. In order to protect the identity of the informants we only provide information of type of informant and city (into brackets).

Qualitative data were categorized to reflect barriers and facilitators for the implementation of universal syphilis screening at first ANC visit and same-day treatment of syphilis-positive women with benzathine penicillin. Barriers and facilitators were grouped into three categories: (1) system-level factors; (2) health providers’ knowledge, practices and skills; and (3) women’s knowledge, preferences, and behavior. Following these findings, we also described the acceptability and feasibility of implementing each intervention component of the PCS Trial and finally, we list the recommendations made to tailor the intervention.

## Results

As shown in Table 1, a total of 112 individuals participated in the formative study: five clinic administrators, 50 health care providers, and 57 pregnant women. Participant characteristics varied between the two countries. While 44 % of the total health care providers were female, in Lusaka the majority were female and in Kinshasa the majority were male. Midwifes and nurses represented 78% of ANC providers in Kinshasa and 31% in Lusaka. The age of providers ranged from 22 to 65 years old in both countries. Overall, around half of the pregnant women interviewed were in their first pregnancy, while in Lusaka the majority were multiparous (69%) and in Kinshasa the majority were primiparous (61%). The median age was 24 and 22 years old, respectively (age range 17–41 in both sites). The vast majority (96%) had completed primary school.

### Barriers and facilitators for testing and treating syphilis at first ANC visit

Table 2 describes factors reported by women, health providers, and clinic administrators that could be barriers and facilitators for syphilis screening and treatment to prevent congenital syphilis. To facilitate understanding, the results are organized into three main sections: first, respondents’ general comments about the current state of practice; second, their thoughts about the feasibility of the PCS Trial’s same-day screening and treatment protocol; and third, their perceptions about the utility, feasibility, and acceptability of the behavioral components of the intervention. Within these overarching categories, we have further stratified the reporting of the results into system- and facility-level factors, provider-level factors, and woman-level factors.

**Table 2:** Barriers and facilitators for same-day screening and treatment in antenatal care to prevent congenital syphilis

Levels	Barriers	Facilitators
System level	• Structural constraints (beyond the health care system) • Fragmentation of the health system • Existence of ANC guidelines in conflict with proposed intervention • Poor accessibility of clinics (geographical and functional) • Staff and product shortages at the PCCs	• Existence of national guidelines for ANC best practice recommendations (including testing and counselling at first visit) • Public health initiatives to improve quality of care
Health providers’ level	• Lack of knowledge and training about evolving best practices • Reservations regarding same-day screening and treatment	• Recognition of importance of early treatment • Willingness to improve methods for testing
Pregnant women level	• Late enrollment in ANC • Lack of knowledge about syphilis, consequences and treatment • Stigma	• Recognized importance of ANC visits • Health education provided at the clinics • Acceptance of same-day screening and treatment at first ANC visit

#### System level

This category covers factors related to the health system that may affect the success of implementing a universal ANC syphilis screening and treatment program. It also includes macro-level factors beyond the scope of the health system, such as the infrastructure and economic situation of countries.

##### Structural constraints that extend beyond the health system

Though discussions of implementation often focus on factors and processes within health facilities, structural constraints can also hinder implementation. For example, interviewees in both countries (but especially in Zambia) noted that electricity power cuts or load shedding can affect the completion of the diagnostic test.

*“There has been an erratic supply of the reagents and so, testing of syphilis has not been very successful… When there is no electricity we have problems because the rotator (Haematocric centrifuge) uses electricity. So, if there is no electricity, the mothers won’t be tested for syphilis and HB that particular day.” (Health provider, − Lusaka)*

In the DRC, it was mentioned that transportation problems make it difficult for health providers to attend clinics on time, contributing to absenteeism.

##### Fragmentation of the health system (DRC-only)

While in Zambia, all PCCs included in the PCS Trial are public and regulated by the government, in the DRC women may seek ANC services from the public or private sectors, with the private sector further subdivided into not-for-profit and for-profit clinics, some of which are not regulated by the government. Several interviewees complained of a *“proliferation of unsustainable private centers,”* given that many private clinics only exist for a short period of time before being shuttered. More generally, these private clinics raise questions about the quality and consistency of care, particularly given that some do not provide all essential components of the ANC package, including PMTCT and syphilis screening, and are not included in government-led quality improvement initiatives.

##### National, NGO policies on ANC and syphilis exist, but conflict with proposed intervention

Interview participants noted that the Ministries of Health in both countries have implemented ANC programs based on the World Health Organization’s best practice recommendations, and health providers are accustomed to following guidelines or protocols to provide care. In addition, they reported that the national guidelines for ANC in both countries included evidence-based approaches for preventing congenital syphilis. In Zambia, all PCCs providing ANC receive guidelines and protocols directly from the Ministry of Health (MOH). Yet in the DRC, the existence of parallel public and private systems has rendered quality control of ANC care complex. Although national ANC guidelines exist, they are not widely distributed to private sector clinics, making it difficult to ensure those providers are kept up to date about best practices.

Interviewees commented that the same-day screening and treatment regimen of the PCS Trial would be a significant departure from established practice, potentially constituting an important barrier to implementation. Current practice and national recommendation in both countries is to provide treatment for syphilis-positive women in a subsequent visit, to which women are encouraged to bring their husband or partner. Moreover, protocols and guidelines promoted by organizations such as International Centre For Aids Care And Treatment Program (ICAP), Centre for Infectious Disease Research in Zambia (CIDRZ), and Elizabeth Glaser Pediatric AIDS Foundation (EGPAF) may conflict with the PCS Trial recommendation to screen and treat at first visit.

That said, providers indicated that they follow protocols for same-day screening and treatment for malaria, worms and anaemia.

*“The first time clients come, we register them to know where they are coming from. Thereafter, we do the palpations, pre-counseling, testing, post-testing, and then give health lessons. After post-counseling, they are given their results. After the results, they get back their antenatal cards.” (Health provider, Lusaka)*

##### Poor accessibility of clinics (geographical and functional)

Interview participants cited multiple ways that access (both physical and financial) constituted barriers to implementation.

Interviewees in both countries mentioned that some women have to travel long distances to access to the clinics.

Interviewees in the DRC also cited cost as a barrier to care. The health system there does not cover the cost of benzathine penicillin, which is passed on to women and their families, creating a cost barrier for women who cannot afford the treatment. Participants also indicated that women’s awareness that not all ANC-related services are consistently covered free of charge may constitute a barrier to them accessing ANC in general, and not just syphilis-related services. However, interviewees in Zambia noted that cost is not a barrier to care there since all test, treatment, and procedures are delivered free of cost to the population.

Another functional barrier is the length of the clinic visit:

*“I think they take much time for the first visit because they have to test a lot of things such as syphilis, BP, and height. The challenge I have is that I have not carried any food. I don’t know whether we are going to finish at 13 or 14 pm, I am starving. Maybe they can adjust a bit, though it is good because they have to know what is happening in your body. I normally wait for 6 hours, it depends on their program.” (Pregnant woman, Lusaka)*

##### Staff and product shortages at the PCCs

Lack of adequate human resources and product shortages at the PCCs also constitute an important barrier to care, according to interviewees.

They noted that staff shortages are a barrier to compliance with established norms and practice guidelines in both countries, given high patient volume in some clinics. In particular, the gender of available providers can have ramifications, as women may feel more comfortable receiving counseling or being attended by female providers.

*“Women who come here are ashamed to undress in front of a male doctor.” (Pregnant woman, Kinshasa)*

Thus, use of female providers in syphilis screening and treatment programs may be a facilitator to uptake and effectiveness. However, there is not agreement on this statement; according to some interviewees (both pregnant women and providers), gender does not affect the patient-provider relationship.

In addition, providers noted that screening and testing products and supplies were not always available. Interviewees were not able to pinpoint exactly why supplies were not available, but indicated that they experienced both general stock-outs and the provision of supplies that they felt were inadequate or subpar. In particular, while some PCCs already used “point-of-care rapid test” (the type test to be provided by the PCS Trial), others did not have access to this test and so used Rapid Plasma Reagin (RPR), which does not provide immediate results.

*“For the time being, the kind of test we are using is the RPR. To me, that is not a rapid test. There is a new way. I don’t know why it is not reaching us. The same technique that is used when conducting an HIV test, is the way even syphilis can be detected. That is a rapid test and is easy to use unlike the other one (RPR test) because it is very difficult for people to read.” (Health provider- Lab technician, Lusaka)*

However, all clinics use point-of-care rapid tests for HIV screening, so providers are familiar with the technology.

#### Health care providers’ level

##### Lack of knowledge and training about evolving best practices

Neither providers nor clinic administrators were aware of a formal system of provider continuing education in either country. As a result, some providers do not have the necessary skills to perform practices included in protocols:

*“We can still call it communication breakdown. For example, we did PMTCT training sometime back in 2009, and of late, I have not seen any refresher course on same and we see protocols every day changing. So if people are not trained, it becomes very difficult to do your job properly. So lack of information updates becomes a challenge especially to us who are implementing on the ground as he said. … So we need more in-house training.” (Health Provider, Lusaka)*

Yet several providers mentioned that they were aware of training clinics and other quality improvement initiatives. However, they noted that often a single representative is chosen to attend the training by the clinic administration, and expressed reservations about the process by which the representatives are chosen.

##### Reservations regarding same-day screening and treatment

Some providers in both countries were not aware that one dose of benzathine penicillin would prevent congenital syphilis. In addition, many exhibited resistance to treating women with benzathine penicillin immediately after screening, citing the importance of treating couples together. They argued that it is not effective to treat women for syphilis without also treating the sexual partner, as the women could be re-infected.

*“When we do RPR, the syphilis test, the strategy is that we encourage the male partners to come with their spouses so that we can test and treat them together as a couple. If we just treat one partner, that problem will come back”. (Health provider, Counselor, Lusaka)*

Health providers also indicated that they believe scheduling a separate follow-up appointment for treating the pregnant woman and her partner improves provision of care. They pointed out that they lack of time to deal with difficult cases (positive women that need counselling and support) when there are many women attending the clinic.

Consequently, providers felt that scheduling a follow-up counseling and treatment visit resulted in higher quality care.

In addition, several health providers in Zambia expressed their concerns over the potential for an adverse reaction to the benzathine penicillin treatment following many hours of fasting.

However, some providers did indicate that they would be willing to provide same-day screening and treatment, and many do recommend treatment on the same day:

*“Providing treatment on the same day is more effective because information about syphilis is still fresh in their minds. If they go without treatment, others may give that as a reason why they don’t go to the clinic.” (Health Provider, Lusaka)*

##### Recognition of importance of early treatment and desire for better methods

Despite the lack of training and skills mentioned, the procedures proposed by the PCS Trial seem easy to understand and similar to other already implemented procedures in clinics, thus acquiring skills would be feasible. The following is a quote from a lay counsellor in Lusaka:

*“Working in antenatal care has been very good experience. Previously, we used to take the rapid tests for HIV testing to the laboratory. But this time around, we do on our own rapid tests by finger pricking, collect the blood and then do the test. We have also come to learn how to do the HB test in antenatal care.” (Health Provider, Zambia)*

*“In some cases, the patient starts crying. And it may take an hour or more to try to calm her into accepting the result.” (Health Provider, Kinshasa)*

#### Pregnant women level

##### Varied perceptions around the importance of ANC

Women usually begin ANC late in pregnancy. Under these circumstances, detection of syphilis in the first trimester of pregnancy is impossible. Some reasons interviewees gave for late initiation of ANC were distance to the clinic, difficulties of organizing childcare, cost of transportation to PCCs and treatment costs. In addition, in the DRC interviewees mentioned that traditional healers and/or local religious leaders discourage the population from attending clinics and promote mistrust of medical treatments.

*A provider claimed that he personally heard a pastor telling his followers that “clinics and hospitals are demonic places. When a child gets sick, you must bring it to the church.” (Health provider, Kinshasa)*

In addition, some women expressed concerns about the safety of the medical treatment.

*“the excessive use of drugs against syphilis can cause the death of the child.” (Pregnant Woman, Kinshasa )*

Still, most women at both sites recognized the importance of ANC for their health and the health of their babies.

*“They teach us everything at the clinic such as how to look after your pregnancy, danger signs in pregnancy, how to live with your husband, preparation for labour, eg, plastics, clamps for baby, vitenge, and nappies.” (Pregnant woman, Lusaka)*

*“In my opinion, we get more advanced information at the clinic than what we learnt at secondary school” (Pregnant woman, Lusaka)*

##### Lack of knowledge about syphilis, consequences and treatment

Generally, women’s level of knowledge about syphilis, and its diagnosis and treatment was low. Some women indicated that they did not know that syphilis could have consequences for their babies before attending ANC.

*“I have heard of syphilis only on Saturday when I came to the clinic.” (Pregnant woman, Kinshasa)*

Most of the interviewed women knew they had been tested for syphilis at the first ANC visit, but there were misconceptions about how syphilis is diagnosed. For example, some women believed that syphilis testing involved a urine sample or vaginal examination. Women also had misconceptions or lacked information regarding the way to treat syphilis to prevent congenital transmission.

Some providers and clinic administrators stated that it is difficult to provide information to women due to high rates of illiteracy. Still, many women reported that they received information about congenital syphilis at the clinics and considered it very useful. They also recognized the importance of being tested and treated for the safety of the baby.

*“The advantage is that they will give you medicine. You will be cured and will also protect the unborn baby. The baby will not suffer from any illness if you take the medication.” (Pregnant woman, Lusaka)*

*“He may even put blame on you that you are positive and that he is not. So it is not necessary to wait for his opinion but the opinion of the health provider.” (Pregnant woman, Lusaka)*

##### Stigma

Providers said that women may have fears of being stigmatized by other community members and may also have fears about communicating with their partner about the infection:

*“[Talking about HIV and sexually transmitted infections,) Stigma comes out as a barrier because much as we go into the community and sensitize the importance of partner notification and partner involvement, we still see that others don’t come as couples to health facilities. As a result, it becomes a challenge for us to implement these strategies. And some people don’t want to be seen at a health facility, so that still becomes a barrier.” (Health provider, Lusaka)*

##### Need for partner consent

There is a shared opinion among some providers that the husband/sexual partner would have a negative influence on women’s decisions to receive treatment on the first visit, if she were to require her partner’s consent. However, the opinions of women were different:

*“I think it is not necessary to wait for your husband because he might refuse if he did not get the information we received” (Pregnant woman, Lusaka)*

### Feasibility of the implementation of the PCS Trial intervention components

The intervention proposed by the PCS Trial seems acceptable for clinic administrators, health providers and pregnant women at the PCCs. The implementation of the intervention is also feasible.

#### Supplies

Acceptability of the point of care rapid tests: In DRC, providers routinely use this kind of tests for syphilis screening in most clinics, but in Zambia the RPR test is used. That said, there are successful experiences regarding the implementation of rapid test for HIV in both countries and the tests are conducted by different providers: lab technicians, midwifes/nurses, and lay counselors. Overall, providers are familiar with rapid tests in both countries.

Acceptability of test and treatment kits: The proposal to have all the supplies needed for the day in boxes at the visit room is well accepted. Respondents are willing to have all the supplies easily accessible.

*All providers said they are willing to “receive syphilis rapid test kits to enable us to work well.” (Health Provider, Kinshasa )*

#### Behavioral interventions

##### Opinion leader strategy

Opinion leaders among prenatal health providers will disseminate, implement, and maintain the intervention using reminders, monitoring, and feedback. The identification and training of opinion leaders was a strategy usually used for other programs at the PCCs level.

The peer selection of these opinion leaders was also well accepted. Providers consider it improvement over the discretionary selection that usually occurs. Measures will be taken to avoid training people that are not motivated to disseminate the intervention within the clinics.

*“Just to add on the training that is coming. If it can be considered to train the right cadres. It is a common trend in these ministries that people will go and be trained, but sit on information without implementing it. So if they can identify and train people that are effective and willing to implement such services.” (Counselor, Lusaka)*

To ensure the success of this component, providers and clinic administrators suggested selecting of opinion leaders who are permanent staff, and implementing incentives for the opinion leaders in order to motivate them to add responsibilities to their current duties.

##### Reminders

Printed material such as posters targeting health providers are well-accepted among health professionals in both settings. Interviewees allowing each clinic to decide on the content of materials (posters) and places to put them to take in account the specific context of the clinic

##### Audit and feedback

Although PCCs may have had some past experience with data-based monitoring and evaluation, we anticipate difficulty in implementing the system of audits and feedback, as PCCs may not have robust data registries or trained personnel who can conduct the required analysis.

##### Women Counseling

Informants suggested including the development of short messages and printed material for pregnant women who test positive in local languages or using images/drawings for illiterate women.

### Tailoring the “PCS Trial” intervention

Based on the findings, we identified specific actions required to tailor each component of the intervention to the two contexts:

- Initially, the planned intervention proposed screening and treatment supplies packed in kits for individual women (including point-of-care rapid test kits for syphilis diagnosis; treatment kits with benzathine penicillin 2.4 MU, syringe and needle, instructions, and information on side-effects; and anaphylaxis treatment kits for emergency use if needed). However, after the formative research it was chosen to use plastic boxes that contained all of the necessary supplies for the day; the boxed supplies could then be replaced the following day.
- The process for selecting opinion leaders was changed from a discretionary selection to be completed using a peer nomination. Consequently, a Hiss questionnaire was adapted to permit peer nomination, and each clinic selected 2–3 opinion leaders.
- Opinion leader training was tailored to address specific needs observed during the formative phase. In particular, training focused on (1) identifying priority health conditions to be screened and treated during pregnancy, (2) showing evidence about the utility of treating women at first visit even though their partner has not been treated yet, (3) reviewing how to screen and manage maternal syphilis, and (4) ensuring optimal stock management.
- Placing Reminders: Simple messages and figures to remind prenatal health providers to conduct the screening procedures and to provide treatment for those women found positive were accepted and suggested to be developed by the selected opinion leaders during the training provided by the study coordinators.
- Incorporation of a supportive supervision component. The initial intervention suggested that a coordinator from the local research teams would meet periodically with each facilitator team to assess the completion of activities and address unexpected problems. However, based on the formative research this was changed to a model of supportive supervision to support the opinion leader while they implement the intervention components at their clinic. A specific guideline for supportive supervision was developed, as well as a direct observation guideline. The supportive supervisor will provide monitoring information (clinic flow charts) that the facilitator can then use for feedback and academic visits.
- Similar projects in the literature recommend including a component to empower the population for their screening and treatment. However, the findings from this study indicated that a patient empowerment component to the PCS Trial would not necessarily influence health provider behavior.

## Discussion

This formative research provided useful information to tailor an intervention intended to prevent congenital syphilis in urban clinics in two African countries. Barriers for the successful implementation of same-day screening and treatment for syphilis at first ANC visit exist at system-, facility- provider- and patient-levels. Findings are similar than the reported in other studies [23–25]. System-level barriers include poor geographical and functional accessibility, lack of human resources and supplies, and established norms that need to be changed in order to implement the new practice, as well as the broader economic situation in these countries. Products and supplies are provided by national programs, NGOs, or international initiatives, but they are frequently depleted. The frequent stock-outs may be due to poor managerial capacities of health workers [26].

Among ANC providers, we identified barriers such as low awareness regarding the evidence of effectiveness of single-dose benzathine penicillin to prevent congenital syphilis, and reservations towards treating women on the first visit. Lack of training of health providers was identified as a barrier to the screening and treatment of pregnant women during ANC, confirming the findings of other authors, such as Watson et al. in Tanzania [24].

From the woman’s perspective, we identified barriers such as late enrollment in ANC due to transportation or cost, and lack of knowledge and information about syphilis and its treatment. As highlighted by other researchers, cost of treatment can be a critical barrier for syphilis screening and treatment, especially for those women who are totally financially-dependent on their male partner [10, 11]. Other studies have also reported that long distances to screening facilities and lack of supplies for syphilis screening and treatment are associated with delay or failure to screen [10, 25, 26].

Given these barriers, promotion of evidence-based practices around syphilis screening and treatment presents a real challenge. However, facilitators of change exist as well. These include the existence of national guidelines currently used at clinics, which recomend syphilis screening at the first ANC visit. Overall, providers seem to have a positive attitude towards improving current practices. In addition, pregnant women understand the importance of being tested, and accept being tested and treated during the same visit if positive.

Findings confirmed that the evidence-based practice of testing for syphilis at the first ANC visit and treating with benzathine penicillin was acceptable to both providers and patients. Regarding the implementation of the PCS Trial intervention strategy, we found that the components included in the intervention were considered acceptable and feasible for implementation at the PCCs. However, the intervention was tailored to better fit the context based on the findings of this study. Specific modifications were proposed in the light of the findings of this formative research. The most substantial change was the addition of a supportive supervision component that consisted of formal support to the opinion leaders from the local intervention coordinator on a monthly basis. Other changes included use of an HISS questionnaire to select the opinion leaders by peer nomination, design of the opinion leader training, and type of reminders. Finally, the formative research indicated how to best pack the products and supplies to fit local needs.

Each of these changes was made to tailor the intervention to the local context, given that – per principles of implementation science – interventions must be right-sized for their implementation contexts in order to be effectively operationalized. Most importantly, in this study, the researchers and implementers worked in close collaboration, making joint decisions about the design of the research intervention, so that the final intervention was informed by implementers’ understanding of what would work in the field.

### Study limitations

As with all qualitative research, findings helped researchers discover and explore themes, generate illuminating and illustrative personal narratives, but they may not be generalizable to the larger population of practitioners or pregnant women in these countries. We were aware about the possibility about obtaining social desirability responses (the wish to appear as a morally-worthy person to the interviewer). In order to minimize this bias in sensitive questions, as for example when asking pregnant women about the influence of their sexual partners regarding the acceptance of treatment if they test positive, we used indirect questioning – asking about what pregnant women in general feels and how they will behave – allowing respondents to project their own feelings onto others.

The study was conducted in urban settings in the capital cities of the countries participating in the study, so the findings may not be relevant for other urban or rural clinics. Findings were based on opinions and self-report rather than observation of actual practices.

Although the sample size in one country was smaller than in the other, findings are consistent across sites and we cover all type of clinics.

## Conclusion

This formative research allowed us to identify factors that may hinder (barriers) or enable (facilitators) the same-day syphilis screening and treatment, and to identify additional, context-specific factors that may influence the implementation of the PCS Trial intervention. Based on those findings we tailored the intervention in order to be feasible.

Interventions that work well in controlled settings may fail during broader roll-out, in part because the controlled settings do not mimic real-world contexts. It is imperative, then, to design interventions from the start with an eye towards the constraints and assets of the context in which they will be implemented. Thus, formative research – research that occurs before an intervention designed and implemented – such as that conducted in this study, is a critical step in designing appropriate and effective interventions. In addition, including implementers and other end-users in intervention design is essential to developing interventions that will be both effective and feasible. These types of best practices are critical to help close the “know-do gap” and speed up progress on ensuring that evidence-based practices reach the populations in greatest need.

## Additional files

Additional file 1: Appendix S1 Preventing Congenital Syphilis Trial. About the Preventing Congenital Syphilis Trial. Summary of the Trial where the Formative Research was implemented. (DOCX 126 kb)

Additional file 2: Appendix S2 DiscussionGroupGuide-Providers. Questionnaire for the interviews with prenatal health providers. Blank, English language version of the interview guide with health providers used in data collection for the study. (DOCX 29 kb)

Additional file 3: Appendix S3 DiscussionGroupGuide-HospitalAdministrators. Questionnaire for In-Depth Interviews with Prenatal Hospital Authorities or Administrators. Blank, English language version of the interview guide with hospital administrators used in data collection for the study. (DOCX 28 kb)

Additional file 4: Appendix S4 DiscussionGroupGuide-PregnantWomen. Questionnaire for focus groups with Pregnant Women. Blank, English language version of the interview guide with pregnant women used in data collection for the study. (DOCX 26 kb)
