# Supplementary material for: Barriers and facilitators to the implementation of antenatal syphilis screening and treatment for the prevention of congenital syphilis in the Democratic Republic of Congo and Zambia: results of qualitative formative research
Source: BMC Health Serv Res. 2017 Aug 14;17:556. doi: 10.1186/s12913-017-2494-7 (PMC5556622; doi:10.1186/s12913-017-2494-7)

### Annex 1: About the “Preventing Congenital Syphilis Trial”

The Preventive Congenital Syphilis Trial (PCS Trial) is a facility-based, two-arm parallel cluster randomized implementation trial in 20 clusters in DRC and Zambia. Clusters were one or more Primary Care Clinics (PCCs) located in the same health area or catchment with expecting number of first ANC visit per year of at least 300 pregnant women.

Zambia is conducting the Congenital Syphillis study in 10 urban PCCs, in the capital city, Lusaka. All of these clinics are public with an expected average number of first ANC visit per clinic per year varies from about 500 to 3200. All of them have implemented the PMTCT program as part of comprehensive ANC.

DRC is including 10 clusters in Kinshasa, each cluster have one to three PCCs representing a total of 16 PCCs. In this country, the type of the selected PCCS are public (n=5), benefit private (n=3) and confessional –supported by a religious organization or none-benefit private (n=8). 14 of the 16 PCCs implement the PMTCT program. Expected average number of first ANC visit per clinic per year varies from about 180 to 900.

Figure 1 shows the PCS Trial design. The trial has two arms, the intervention group *(“SuppliesPlus”) and* control group *(“Supplies”)*. For 18 months, the prenatal clinics in the intervention clusters will conduct the behavioral multifaceted intervention. The prenatal clinics in the control clusters will continue their usual in-service activities. Supplies for screening and treatment will be provided to both intervention and control groups.

The behavioral intervention is based on the stages of change and organizational change theories and will be tailored by formative research. It consists of a multifaceted intervention with several interacting components to increase the use of evidence-based practices in prenatal care visits, such as screening and treatment for syphilis, and will include:

- Identification and training of opinion leaders among prenatal health providers, who will disseminate, implement, and maintain the best evidence practices using reminders, monitoring and feedback;
- Packaging the supplies in kits:
  - Point-of-care rapid test kits for syphilis diagnosis with instructions for immediate treatment, if positive;
  - Treatment kits (Benzathine Penicillin 2.4 MU, syringe and needle, instructions, and information on side-effects);
  - Anaphylaxis treatment kits for emergency use if needed (a kit containing resources to treat an anaphylaxis adverse reaction according to local practice guidelines).

Figure 1: Preventive Congenital Syphilis Trial (PCS Trial) design.


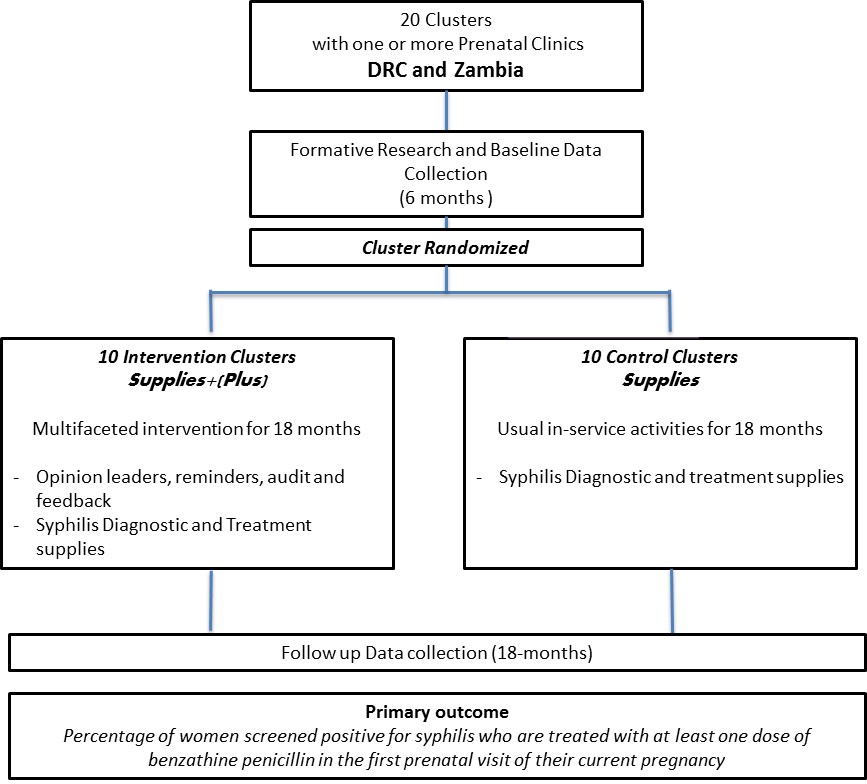

Supplement: Supplementary file 1 — Appendix S1 Preventing Congenital Syphilis Trial. About the Preventing Congenital Syphilis Trial. Summary of the Trial where the Formative Research was implemented. (DOCX 126 kb) [file 12913_2017_2494_MOESM1_ESM.docx]
