# Supplementary material for: Barriers and facilitators to the implementation of antenatal syphilis screening and treatment for the prevention of congenital syphilis in the Democratic Republic of Congo and Zambia: results of qualitative formative research
Source: BMC Health Serv Res. 2017 Aug 14;17:556. doi: 10.1186/s12913-017-2494-7 (PMC5556622; doi:10.1186/s12913-017-2494-7)
Supplement: Supplementary file 2 — Appendix S2 DiscussionGroupGuide-Providers. Questionnaire for the interviews with prenatal health providers. Blank, English language version of the interview guide with health providers used in data collection for the study. (DOCX 29 kb) [file 12913_2017_2494_MOESM2_ESM.docx]

**GROUP DISCUSSIONS WITH PRENATAL HEALTH PROVIDERS**

***Question Guide***

*INTRODUCTORY QUESTIONS*

| 1 | How long have you been working at this prenatal clinic? |
| --- | --- |
| 2 | What is your current position at this prenatal clinic? How long have you been in this position? |
| 3 | What are your main responsibilities? |

*PRENATAL CARE PROCESS*

I'm going to ask you to describe for me the process of prenatal care from the moment a woman reaches the health care center for her routine visits, until she leaves the prenatal clinic.

| 4 | Who is involved in her health care? |
| --- | --- |
| 5 | Which are your responsibilities and which are the responsibilities of other professionals involved? |
| 6 | When do you come into contact with the woman (from the time she arrives at the clinic until she leaves)?  How much time do you usually spend with a woman during a visit? |

*HEALTH PROMOTION STRATEGIES*

Usually, during antenatal care, some health promotion activities are implemented...

| 7 | Is there any strategies currently being implemented? |
| --- | --- |
| 8 | Would you please name which activities are currently being implemented?   - - What do they consist of?   - Who are they targeted to?   - Who promotes new health policies/recommendations targeted to pregnant women? (Health Ministry/international agencies/health care facility director)   - Which professionals (nurses, midwives, or physicians) are in charge of implementing the new health policies/recommendations?   - Why? |
| 9 | What is your role in the implementation of these polices? |

Some health promotion strategies to adopt new policies/recommendations during antenatal care turn out to be successful, in the sense that the professional staff adhere to their implementation so that a large number of women start receiving that intervention or advice…

| 10 | Could you name any strategy you remember being particularly successful?   - - Who promoted it?   - What did it consist of?   - Who implemented it?   - How was it implemented? |
| --- | --- |
| 11 | Why do you think it was successful? Are there aspects of this strategy (or other strategies) that were not successful? Why? |
| 12 | Can you name any health promotion strategy that was not successful? Why? |
| 13 | What do you think are the main barriers to adopt new recommendations in health care? |
| 14 | In what ways does the person promoting the strategy affect its success? |
| 15 | What kinds of materials are usually used in health promotions? |
| 16 | With which ones do you feel most comfortable? |
| 17 | Which ones do you consider are the most effective? (in the sense that they achieve the greatest impact)   - Why? |

*CURRENT PRACTICE*

As it is well known, syphilis has implications for women’s and newborn’s health. According to recent figures, the seroprevalence of women with syphilis among prenatal care attendees was estimated to be ……% in your country. Likewise, we know that syphilis is often stigmatized and of low priority, and women are often not attending prenatal care or are attending late …

| 18 | Have you ever received any formal education on how to test a pregnant woman for syphilis? |
| --- | --- |
| 19 | What is your usual practice regarding screening for syphilis in prenatal care? |
| 20 | What kinds of tests do you know about to screen for syphilis?  Are you familiar with the available tests in your clinic?  Are there available rapid tests to use in your clinic?  Which tests do you use routinely? |
| 21 | Do you treat a woman on the same day that you perform the test?  Are there drugs available for treatment?  If you cannot treat the woman when you know she is infected, how do you follow up with her? |
| 22 | Are there other health professionals involved in the screening and treatment process for syphilis? |
| 23 | Do women generally accept to be tested and treated? |

*INCORPORATION OF HEALTH CARE PROMOTION STRATEGIES*

If you think about incorporating a new health promotion strategy or increasing the use of an existing practice during antenatal care that would have to be provided to all women you care for at the first visit...

| 24 | Could you think of aspects that would facilitate its inclusion and effective implementation?   - Who should be in charge of implementing it? Why? - Who should support it? - At what moment should it be included? - What would make the implementation of a health promotion strategy easier without creating resistance? |
| --- | --- |
| 25 | Can you tell what you consider to be the potential barriers that would affect the incorporation of a strategy? |

*INTERVENTION*

We are carrying out a research project to evaluate a behavioral intervention to increase the frequency of pregnant women who are screened for syphilis at their first prenatal visit and immediately treated if infected, compared to providing supplies only.

We seek to show that combining the provision of supplies with a multifaceted behavioral intervention is more effective than providing supplies only.

We are planning to package the following supplies in kits:

- Point-of-care rapid test kits for syphilis diagnosis with instructions for immediate treatment, if positive;
- Treatment kits (benzathine penicillin 2.4 MU, syringe and needle, instructions, and information on side-effects);
- Anaphylaxis treatment kits for emergency use if needed (a kit containing resources to treat an anaphylaxis adverse reaction according to local practice guidelines).

| 26 | Can you tell me what the consequences of maternal syphilis are on an infant’s health? |
| --- | --- |
| 27 | What are the benefits of early treatment of syphilis during pregnancy? |
| 28 | What are the harms of treatment for syphilis during pregnancy? |
| 29 | Do you know what messages might be most effective in pregnant women to encourage testing and treatment for syphilis as soon as possible? |
| 30 | Do women attend prenatal care in early stages of pregnancy?  How many visits do you estimate women attend? |
| 31 | What do you think is the best way to test and treat a woman for syphilis in the first visit? |
| 32 | Which professional do you think should implement this strategy? (OB/GYN, midwife, nurse)   - Why?   Do you think this person could implement both steps of the intervention? Testing and treatment? |
| 33 | When, during prenatal care, do you believe that this strategy would achieve its greatest impact? |
| 34 | Do you believe it would be feasible to implement this intervention in a similar health care facility?  How much time do you think you could be spend on this? |
| 35 | What would be the facilitators to incorporate this strategy? |
| 36 | What would be the barriers to incorporate this strategy? |
| 37 | What aspects would facilitate the inclusion of this strategy during the prenatal care visit? |
| 38 | What other aspects do you think we should consider regarding this issue? |
| 39 | What do you think about having point-of-care rapid test kits for syphilis diagnosis, with instructions for immediate treatment, in prenatal clinics? |
| 40 | What kind of package do you imagine would be most feasible to incorporate this intervention systematically in every first visit? |

*IMPLEMENTATION*

Now, let me tell you the mechanism that we have designed to successfully incorporate this strategy for increased screening for syphilis.

First, we are planning to identify and train a group of professionals who will be responsible for disseminating the strategy among those health care professionals who are in charge of attending women, to include using the rapid test and treating a woman if she is infected.

We are planning to identify this group through a peer nomination process, which aims to identify people who are generous with their knowledge, have experience in their work, and are respected by their peers. We will use a questionnaire for the peer nomination process.

| 41 | Who do you think should be included in this group to disseminate the strategy to other health professionals? (OB/GYN, midwife, nurse)   - Just one type of professionals or a mixture? - Why? |
| --- | --- |
| 42 | What do you think about this selection strategy? |

As I already mentioned, this group would receive a specific one and half-day training and then create a plan to disseminate their knowledge among the rest of their team to try to achieve the incorporation of the intervention....

| 43 | What kind of training do you think might be necessary to provide for those professionals who will be responsible for implementing this strategy? |
| --- | --- |
| 44 | What activities should be undertaken by trained professionals?  How would the trained professionals disseminate their knowledge among the rest of the team? Would the rest of the team be supportive of the initiative? |

The involvement of trained professionals would require the use of a rapid test…

| 45 | Do you think it would be feasible to use rapid tests in the first visit for screening syphilis? |
| --- | --- |
| 46 | How do you think this group would take the responsibility to perform the test and to inform the woman of the results? |
| 47 | How do you think this group would take the responsibility to treat those woman whose tests are positive? |
| 48 | Would it be necessary to involve any strategy in order to increase the project´s feasibility?   - How? |
| 49 | Do you have any final thoughts or comments about use of rapid tests and treatment point of care? |

The involvement of the trained professionals might require a greater investment of time in the health center...

| 50 | Do you think it would be feasible to ask professionals to spend an extra several minutes to test every pregnant woman for syphilis in their first visit? |
| --- | --- |
| 51 | How do you think this group would take that responsibility? |
| 52 | Would it be necessary to involve any strategy in order to increase the project´s feasibility?   - How? |
| 53 | Do you have any final thoughts or comments? |
