# Supplementary material for: Barriers and facilitators to the implementation of antenatal syphilis screening and treatment for the prevention of congenital syphilis in the Democratic Republic of Congo and Zambia: results of qualitative formative research
Source: BMC Health Serv Res. 2017 Aug 14;17:556. doi: 10.1186/s12913-017-2494-7 (PMC5556622; doi:10.1186/s12913-017-2494-7)
Supplement: Supplementary file 3 — Appendix S3 DiscussionGroupGuide-HospitalAdministrators. Questionnaire for In-Depth Interviews with Prenatal Hospital Authorities or Administrators. Blank, English language version of the interview guide with hospital administrators used in data collection for the study. (DOCX 28 kb) [file 12913_2017_2494_MOESM3_ESM.docx]

**IN-DEPTH INTERVIEWS WITH PRENATAL HOSPITAL AUTHORITIES OR ADMINISTRATORS**

***Question Guide***

*INTRODUCTORY QUESTIONS*

| 1 | How long have you been working at this prenatal clinic? |
| --- | --- |
| 2 | What is your current position in this prenatal clinic? How long have you been in this position? |
| 3 | What are your main responsibilities? |
| 4 | Do you still attend patients in this prenatal clinic? |

*INCORPORATION OF HEALTH CARE PROMOTION STRATEGIES*

We are interested in learning how health promotion strategies for pregnant women are incorporated and carried out at public health care centers; particularly, how prenatal clinic policies and recommendations influence this. We will start with some general questions…

| 5 | Who promotes new health promotion strategies targeted to pregnant women?   - Health Ministry/international agencies/health care facility director |
| --- | --- |
| 6 | Where do you usually obtain information about current recommendations on perinatology? Who do you talk to when you want information about new clinical practices? |
| 7 | How do you decide to incorporate new health promotion strategies at this facility? Are there specific criteria used in making this determination? What are the current health promotion strategies in place? Do you have written protocols? |
| 8 | How do you communicate decisions/new protocols to the health staff? Once you have decided to promote changes to a particular clinical practice, how do you communicate that to the health staff?  How do you monitor if the changes actually occur? |
| 9 | Which professionals (nurses, midwives, or physicians) are in charge of implementing new health promotion strategies?   - Why? Is it part of their job description or do they volunteer? Are there any benefits to staff in implementing these programs? Are there any drawbacks to staff? - Do you think it is effective this way? |
| 10 | How do you monitor if the strategies are implemented? And implemented well? |
| 11 | Do you consider it important to establish a mechanism for patients to express their opinions about services received in the prenatal clinic? Why? |
| 12 | Does your prenatal clinic have a mechanism with that objective? How does it work?  If it doesn’t exist, how would you implement it? |

*CURRENT PRACTICE*

As it is well known, syphilis has implications for women’s and newborn’s health. According to recent figures, the seroprevalence of women with syphilis among prenatal care attendees was estimated to be ……% in your country. Likewise, we know that syphilis is often stigmatized and of low priority, and women are often not attending prenatal care or are attending late …

| 13 | Does this facility have any routine policy to test for syphilis among pregnant women in their first visit?   - If yes, please describe. If no, what is the reason? |
| --- | --- |
| 14 | Are providers given any formal or informal training regarding preventing congenital syphilis?   - What is the training? From whom do they receive the training? - How important is it to you that providers address prenatal screening? |
| 15 | Are you using a rapid test to screen for syphilis? Please explain what kinds of tests you use and who provides them. |
| 16 | Do you use a standard clinical record?   - Does it include screening and treatment for syphilis? - Would it be possible to document screening and treatment for syphilis for women in their clinical record or other documentation? |

*INCORPORATION OF HEALTH CARE PROMOTION STRATEGIES*

If you think about incorporating a new health promotion strategy or increasing the use of an existing practice during antenatal care in the first visit…

| 17 | Could you think of aspects that would facilitate its inclusion and effective implementation?   - Who should be in charge of implementing it? Why? - Who should support it? - At what moment should it be included? - What would make the implementation of a health promotion strategy easier without creating resistance? |
| --- | --- |
| 18 | Can you think of aspects that would affect the incorporation of a strategy?   - Who should not be in charge? Why? - At what moment should it not be included? - What would generate resistances in incorporating a health promotion strategy? |

*INTERVENTION*

We are carrying out a research project to evaluate a behavioral intervention to increase the frequency of pregnant women who are screened for syphilis at their first prenatal visit and immediately treated, if infected, compared to providing supplies only.

We seek to show that combining the provision of supplies with a multifaceted behavioral intervention is more effective than providing supplies only.

We are planning to package the following supplies in kits:

- Point-of-care rapid test kits for syphilis diagnosis with instructions for immediate treatment, if positive;
- Treatment kits (benzathine penicillin 2.4 MU, syringe and needle, instructions, and information on side-effects);
- Anaphylaxis treatment kits for emergency use if needed (a kit containing resources to treat an anaphylaxis adverse reaction according to local practice guidelines).

| 19 | Can you tell me what the consequences of maternal syphilis are on an infant’s health? |
| --- | --- |
| 20 | What are the benefits of early treatment of syphilis during pregnancy? |
| 21 | What are the harms of treatment for syphilis during pregnancy? |
| 22 | Do you know what messages might be most effective in pregnant women to encourage testing and treatment for syphilis as soon as possible? |
| 23 | Do women attend prenatal care in early stages of pregnancy?  How many visits do you estimate women attend? |
| 24 | What do you think is the best way to test and treat a woman for syphilis in the first visit in your clinic? |
| 25 | Which professional do you think could successfully implement this strategy? (OB/GYN, midwife, nurse)   - Why?   Do you think this professional could implement all steps of the intervention? Do you think this person would receive the support of other professionals? |
| 26 | When, during the prenatal care visit, do you think that this intervention would be most feasible and achieve its greatest impact? (During weight control? Prior to the OB/GYN visit, during the prenatal exam, in a specific place...)  Why? |
| 27 | Do you think this intervention will help women during their prenatal care? |
| 28 | Do you believe this intervention could be implemented systematically in your health care facility? A similar health care facility? |
| 29 | What would be the facilitators to incorporate this strategy? |
| 30 | What would be the barriers to incorporate this strategy? |
| 31 | What aspects would facilitate the incorporation of this strategy during the prenatal care visit? |
| 32 | What other aspects do you think we should consider regarding this issue? |
| 33 | What do you think about having point-of-care rapid test kits for syphilis diagnosis, with instructions for immediate treatment, in prenatal clinics? |
| 34 | What kind of package do you imagine would be most feasible to incorporate this intervention systematically in every first visit? |

*IMPLEMENTATION*

Now, let me tell you the mechanism that we have designed to incorporate this intervention strategy for increased screening for syphilis.

First, we are planning to identify and train a group of professionals who will be responsible for disseminating the strategy among the rest of the health care professionals who are in charge of attending women, to include using the rapid test and treating a woman if she is infected.

We are planning to identify this group through a peer nomination process, which aims to identify people who are generous with their knowledge, have experience in their work, and are respected by their peers. We will use an anonymous questionnaire for the peer nomination process.

| 35 | Who do you think should be included in this group to train other health professionals on how to implement the intervention in a facility like yours? (OB/GYN, midwife, nurse)   - Just one type of professional or a mixture? - Why? |
| --- | --- |
| 36 | What do you think about the peer nomination selection strategy? |

The involvement of the trained professionals would require the use of a rapid test…

| 37 | Do you think it would be feasible to use rapid tests in the first visit for screening syphilis in this prenatal clinic? |
| --- | --- |
| 38 | How do you think providers would take the responsibility to perform the test and to inform the women of the results? |
| 39 | How do you think providers would take the responsibility to treat women whose tests are positive? |
| 40 | Would it be necessary to involve any strategies in order to increase the project´s feasibility?   - How? |
| 41 | Do you have any final thoughts or comments about point-of-care rapid test kits for syphilis diagnosis with instructions for immediate treatment? |

The involvement of the trained professionals could involve a greater investment of time in the health center...

| 42 | Do you think it would be feasible to ask professionals to spend an extra several minutes to test every pregnant woman for syphilis in their first visit? |
| --- | --- |
| 43 | How do you think this group would take the responsibility? |
| 44 | Do you have any final thoughts or comments? |
