# Supplementary material for: Barriers and facilitators to the implementation of antenatal syphilis screening and treatment for the prevention of congenital syphilis in the Democratic Republic of Congo and Zambia: results of qualitative formative research
Source: BMC Health Serv Res. 2017 Aug 14;17:556. doi: 10.1186/s12913-017-2494-7 (PMC5556622; doi:10.1186/s12913-017-2494-7)
Supplement: Supplementary file 4 — Appendix S4 DiscussionGroupGuide-PregnantWomen. Questionnaire for focus groups with Pregnant Women. Blank, English language version of the interview guide with pregnant women used in data collection for the study. (DOCX 26 kb) [file 12913_2017_2494_MOESM4_ESM.docx]

**GROUP DISCUSSIONS WITH PREGNANT WOMEN**

***Question Guide***

*INTRODUCTORY QUESTIONS*

| 1 | Is this your first child? |
| --- | --- |
| 2 | What are the things about motherhood that excite you? |
| 3 | Have you done anything to prepare the coming of your baby? |
| 4 | Were you ever attended at this center before? |
| 5 | When did you start prenatal care at this center? |
| 6 | Is this health center near your house? |
| 7 | How many prenatal care visits have you had so far? |
| 8 | How long do you have to wait each time you come to this center for prenatal care? |
| 9 | How does a usual prenatal visit develop? (What happens from the time you arrive until you leave?) (Learn about the nursery, physician, waiting times, etc.) |
| 10 | Do you feel comfortable talking to any of your health care providers about your health? If yes, who? If no, why?? (nurses, physicians, midwifes) |
| 11 | With which health professional do you spend more time? |
| 12 | What is your main source for health information? |

*PREVENTION STRATEGIES*

Usually, some health promotion activities are implemented at health care facilities during prenatal care. Health care promotion activities can refer to test that is given to promote healthy life styles, as well as activities undertaken by health professionals that promote health and prevent diseases. For example: tests to prevent complications to your health and your baby’s health, like syphilis, anemia, HIV, proteinuria, etc.

| 13 | Can you identify if these kinds of tests were given to you to prevent some disease/problem during pregnancy or to promote a healthier pregnancy? |
| --- | --- |
| 14 | If yes, what were the tests that you received? |
| 15 | Within the health care team, who was the person responsible for asking you about the tests? |
| 16 | At what moment did he/she perform these procedures? (When you arrived, during the visit, when you were leaving, etc.) |
| 17 | Which is the test you most remember, and why do you think it made a higher impact on you? |
| 18 | Were you given any kind of written information (i.e., brochures) about these promotion activities? How was this information presented? Was it clear? |

*PREVIOUS KNOWLEDGE ABOUT SYPHILIS*

We are testing a behavioral strategy to increase the number of women who receive screening for syphilis to prevent congenital syphilis in their babies.

| 19 | What do you know or what have you heard about syphilis during pregnancy? |
| --- | --- |
| 20 | What do you know about the health consequences of syphilis during pregnancy on the health of your baby and on your health? |
| 21 | What are the benefits of being diagnosed during early pregnancy for you? For your baby? |
| 22 | What are the harms of being diagnosed during early pregnancy for you? For your baby? |
| 23 | Have you been tested for syphilis during a routine prenatal care visit during this pregnancy?   - How were you tested? - How did you feel about it? - What do you consider to be the best way to do this screening? |
| 24 | If your test was positive, would you agree to receive treatment in the same visit? |
| 25 | Have you ever received any written information about this issue? (brochures, triptychs, etc.) |
| 26 | What do you think are the most important messages for someone like you to communicate about benefits to being tested for syphilis and receiving treatment if a woman is positive? |
| 27 | Would you depend on your husband’s opinion to accept being tested and treated for syphilis? |
| 28 | Do you know of any method that helps you to know if you have syphilis?  Have you ever heard about any drug that cures syphilis?  If a drug like that existed, would you like to receive it if you had a positive test? |

*CURRENT INTERVENTION*

As I told you, we are carrying out a research project to evaluate a behavioral intervention to increase the frequency of pregnant women who are screened for syphilis at the first prenatal visit and who are immediately treated if infected.

Prenatal health providers will ask women to obtain blood by finger prick (100µl or five drops). With the blood, a rapid test for syphilis will be performed; if the result is positive, women will be provided with the option to receive rapid point-of-care treatment via one injection of benzathine penicillin. This might cause pain or soreness at the injection site. In very rare cases, women might have an allergic reaction to the treatment, which will be treated according to the usual practices in the healthcare facility women are attending.

We are planning to package the needed supplies in kits with reminders for the providers.

| 29 | What do you think about this strategy?   - What do you think about this plan? |
| --- | --- |
| 30 | What do you think would be the best way for health professionals to ask you to take the test? |
| 31 | Who do you think should test and eventually treat you?   - Why? |
| 32 | When do you think you should be tested and eventually treated?   - Why? |

If this intervention is implemented at the health care center, this might require several extra minutes of your time…

| 33 | How do you think women would respond to the fact that the prenatal care visit would take several minutes longer than usual in order to be tested? |
| --- | --- |
| 34 | Finally, do you think that this intervention should consider any other aspects that we are not taking into account? |

*WRAP-UP*

Now I am going to ask you to complete this form with some demographic information. The information will be kept anonymous and confidential.

Before you leave, I am going to give you a sheet with information about preventing congenital syphilis during pregnancy. If you have any questions or doubts, you can stay and we will discuss them.

Thank you very much for collaborating with this research.
